# The Solute Carrier Transporter SLC15A3 Participates in Antiviral Innate Immune Responses against Herpes Simplex Virus-1

**DOI:** 10.1155/2018/5214187

**Published:** 2018-07-05

**Authors:** Longzhen He, Baocheng Wang, Yuanyuan Li, Leqing Zhu, Peiling Li, Feiyan Zou, Lianghua Bin

**Affiliations:** ^1^The First Affiliated Hospital, Biomedical Translational Research Institute, The International Immunology Center and The Key Laboratory of Antibody Engineering of Guangdong Province, Jinan University, Guangzhou, Guangdong Province, China; ^2^Department of Developmental and Regenerative Biology, College of Life Science and Technology, Jinan University, Guangzhou, Guangdong Province, China; ^3^Department of Pediatric, National Jewish Health, Denver, CO, USA

## Abstract

The innate immune response is the first line defense against viral infections. Novel genes involved in this system are continuing to emerge. SLC15A3, a proton-coupled histidine and di-tripeptide transporter that was previously found in lysosomes, has been reported to inhibit chikungunya viral replication in host cells. In this study, we found that SLC15A3 was significantly induced by DNA virus herpes simplex virus-1(HSV-1) in monocytes from human peripheral blood mononuclear cells. Aside from monocytes, it can also be induced by HSV-1 in 293T, HeLa cells, and HaCaT cells. Overexpression of SLC15A3 in 293T cells inhibits HSV-1 replication and enhances type I and type III interferon (IFN) responses, while silencing SLC15A3 leads to enhanced HSV-1 replication with reduced IFN production. Moreover, we found that SLC15A3 interacted with MAVS and STING and potentiated MAVS- and STING-mediated IFN production. These results demonstrate that SLC15A3 participates in anti-HSV-1 innate immune responses by regulating MAVS- and STING-mediated signaling pathways.

## 1. Introduction

The innate immune response against viral infection is initiated by pattern recognition receptors (PRR) that recognize virally derived pathogen-associated molecular patterns (PAMPs). Viral nucleic acids, including DNA and RNA, are classic PAMPs that activate signaling pathways leading to induction of type I/type III interferons and proinflammatory cytokines. These cytokines subsequently induce a wide range of genes to restrict and kill invading viruses, mediate inflammatory responses, and regulate adaptive immune responses [1–3].

Cytosolic RNA sensors and DNA receptors have been identified to recognize virus-derived double-stranded RNA and DNA in host cells, respectively. Two DExD/H-box RNA helicases, retinoic acid-inducible gene 1 (RIG-1) and melanoma differentiation-associated gene 5 (MDA5), recognize double-stranded RNA and activate downstream signaling cascades through adaptor MAVS (VISA) [4–6]. A number of cytosolic DNA receptors are reported, including cyclic guanosine monophosphate-adenosine monophosphate (cGAMP) synthase (cGAS) [7], DNA-dependent activator of IFN regulatory factor [8], DDX41 [9], and IFN-*γ*-inducible protein (IFI16) [10]; however, only cGAS has been validated to recognize DNA ligand in various systems both *in vitro* and *in vivo* [11]. STING (MITA) is the adaptor for cGAS to activate IFN responses [12–14].

*SLC15A3* encodes the protein of peptide histidine transporter 2 (PHT2), which is a member of proton-coupled oligopeptide transporters [15]. Mammalian SLC15A3 was first cloned using a rat cDNA library. Its mRNA was abundant in lymphatic tissues of the spleen and thymus. Ectopic expression of SLC15A3 protein was found on the lysosomal membrane. Its function was proposed to transfer histidine and dipeptide out from lysosome to cytosol [16]. Human SLC15A3 was reported to be induced by LPS in macrophages, as well as induced by TNF*α* and IFN*γ* in prostate cell lines [17, 18]. Previous study by Schoggins et al. found that overexpression of *SLC15A3* inhibited chikungunya virus replication in human STAT1-deficient fibroblasts, indicating its involvement in antiviral response [19]. Additionally, SLC15A3 was found to mediate the egress of bacterially derived muramyl dipeptide (MDP) from endolysosome to cytosol in dendritic cells and macrophages, where nucleotide-binding oligomerization domain containing 2 then has a chance to detect MDP and to be activated [20].

In this study, we investigated the role of SLC15A3 in antiviral responses against the DNA virus, herpes simplex virus-1 (HSV-1). Our results demonstrate that SLC15A3 is induced by viral infection to participate in intracellular RNA and DNA receptor-mediated interferon production.

## 2. Methods and Materials

### 2.1. Peripheral Blood Mononuclear Cell Isolation and Purification of Different Cell Types

5 healthy adults were recruited to participate in this study to donate venous blood. The institutional review board at the First Affiliated Hospital of Jinan University approved the study, and all subjects provided written informed consent to participate. Human PBMCs were isolated using Ficoll-Hypaque® density gradient centrifugation of heparinized venous blood from donors. T cells, NK cells, B cells, monocytes, and dendritic cells were then purified from PBMCs using anti-CD3, anti-CD56, anti-CD14 microbeads, and a human B cell isolation kit II according to the manufacturer's guidelines (Miltenyi Biotec Inc., San Diego, CA). PBMCs and other types of cells were maintained in RPMI 1640 supplemented with 10% fetal bovine serum, penicillin (50 I.U./ml), and streptomycin (50 *μ*g/ml) and treated with HSV-1 at different multiplicity of infection (MOI) for 24 h. RNA and protein were harvested from the treated cells.

### 2.2. Cell Lines and Viruses

293T, HeLa cells, Vero cells, and HSV-1 were purchased from ATCC. HaCaT cell line was a gift from Dr. Donald Leung lab (National Jewish Health, Denver, CO). 293T, HeLa, HaCaT, and Vero cells were cultured in DMEM with 10% fetal bovine serum, penicillin (50 I.U./ml), and streptomycin (50 *μ*g/ml) at 37°C with 5% CO_2_. The different types of cells were seeded in 24-well dishes at 1 × 10^5^ and treated with HSV-1 at different MOI for 24 h then harvested for RNA extraction, qRT-PCR, and Western blot assays.

### 2.3. Expression Plasmids, Antibodies, and Cell Organelle Trackers

Myc-DDK1-tagged SLC15A3, MAVS, STING, pCMV6-AC-GFP-SLC15A3, and pCMV6-AN-HA were purchased from OriGene Technologies (Rockville, MD). HA-tagged SLC15A3 was constructed by standard molecular biology techniques. RFP-PXMP2 was a generous gift from Dr. Hong-Bing Shu (Wuhan University, China). Rabbit anti-human SLC15A3 and anti-human Rab11 were purchased from Abcam. Anti-human Rab5 and Rab7 were purchased from Santa Cruz. Alexa Fluor 594 horse anti-mouse or rabbit secondary antibodies and Alexa Fluor 488 goat anti-mouse or rabbit IgG (H + L) antibody were purchased from Thermo Fisher Scientific. Anti-human *β*-actin, anti-Flag, and anti-HA antibodies were purchased from Sigma-Aldrich. Lyso Tracker™ Red DND-99 and ER-Tracker™ Red (BODIPY™ TR Glibenclamide) were purchased from Invitrogen (Carlsbad, CA).

### 2.4. Overexpression, siRNA-Mediated Knockdown, Viruses, and PRR Treatment

Poly(I:C)(HMW)/LyoVec™, Poly(dA:dT)/LyoVec™, imiquimod (IMQ), 2′3′-cGAMP, and HSV60 (5′-TAAGACACGATGCGATAAAATCTGTTTGTAAAATTTATTAAGGGTACAAATTGCCCTAGC-3′) were purchased from Invivogen. 293T cells were seeded in 48-well dishes at 5 × 10^4^/well. The following day, cells were transfected with mammalian expression plasmids of SLC15A3-GFP or HA-SLC15A3 and control empty plasmids. In some cases, the cells were transfected with siRNA duplexes to knockdown SLC15A3 gene expression. The sequences of SLC15A3 siRNA were included in Table S1. The siRNA duplexes were transfected into cells using lipofectamine 2000 according to the manufacturer's guideline (Invitrogen). After 24 h of incubation, the cells were then treated with HSV-1 and different PRR agonists for additional 24 h. The cells were then harvested for RNA extraction and qRT-PCR. The culture supernatants were collected for ELISA. In some cases, the cells and supernatants were harvested together for viral plaque assays.

### 2.5. Immunofluorescence Microscopy

HeLa cells were seeded in cover slips at 60–80% confluence. The following day, cells were transfected with empty plasmids and SLC15A3-GFP, or SLC15A3-GFP and RFP-PXMP2 were cotransfected. To label intracellular organelles, cells were incubated with respective trackers for 24 h, and the cells were then fixed with 4% (*w/v*) paraformaldehyde in PBS for 30 min and treated with 0.1% Triton X-100 in PBS for 10 min. After washing with PBS three times, the cells were treated with 2% BSA in PBS for 30 min to block nonspecific protein binding. For immunostaining, primary antibodies of Rab5, Rab7, or Rab11 in 1% BSA-PBS were overlaid on to the cover slips for 45 min, and the cells were washed three times with PBS and then incubated with secondary antibodies for 45 min. Nuclei were stained with 4,6-diamidino-2-phenylindole (DAPI). Images were taken using a Leica DM6000 microscope (Wetzlar, Germany).

### 2.6. Total RNA Extraction and Real-Time PCR

Total RNA was extracted from cells using Easypure RNA Kit (TransGen Biotech, Beijing, China). High-fidelity cDNA was generated using PrimeScript™ RT Reagent Kit (Takara Bio, Japan). Real-time PCR reactions were conducted using a Bio-Rad CFX system. The primers were synthesized from Sangon Biotech (Shanghai, China). Primer sequences of *SLC15A3*, HSV-1 *gD* gene, IFN-*β*, IL29, and *β*-actin as listed in Table S2. Relative mRNA levels of target genes were normalized by *β*-actin using the ΔΔCT method.

### 2.7. ELISA

Human IL29 (IFN lambda 1) ELISA Ready-SET-Go! kit and human IFN*β* ELISA Ready-SET-Go! kit were purchased from eBioscience. IL29 and IFN*β* proteins in the culture supernatants were measured according to the manufacturer's instructions.

### 2.8. Coimmunoprecipitation and Western Blotting

293T were cotransfected with HA-SLC15A3 expression plasmids and Flag/DDK1-tagged cGAS, MAVS, STING, DDX41, and MyD88 using Lipofectamine 2000. After an overnight incubation, cells were lysed in protein lysis buffer (20 mM Tris-HCl pH 7.4, 150 mM NaCl, 1 mM EDTA, and 1% Triton X-100) supplemented with protease inhibitors. Protein lysates were incubated with 0.5 *μ*g of anti-HA antibody and 30 *μ*l of protein A/G plus agarose (Santa Cruz Biotechnology) in 1.5 ml Eppendorf tubes and rotated at 4°C for one hour. The beads were then washed three times using protein lysis buffer with 0.5 M NaCl. After washing, 30 *μ*l of the 2x Laemmli sample buffer (Bio-Rad) was added to the beads and then boiled for 5 minutes. The supernatants from each sample were then subjected to Western blotting for detection of either Flag/DDK1-tagged protein or HA-tagged protein. A standard Western blot protocol was used.

### 2.9. Viral Plaque Assay

Vero cells were seeded into 24-well dishes at 2 × 10^5^ to form monolayers. HSV-1-infected cells were frozen and thawed three times; serial dilutions were then made and added to Vero cell monolayers. Cells were then incubated for 2 h to allow viral absorption. After the incubation, infectious media was removed; the cells were then covered by 0.2% of agarose made with DMEM containing 2% fetal bovine serum and cultured at standardized cell culture condition. 4 days later, the viral plaque formation was visualized by 1% crystal violet staining.

### 2.10. Statistical Analysis

The statistical analyses were conducted using GraphPad prism, version 6.01 (San Diego, CA). Comparisons of expression levels were performed using ANOVA techniques and independent sample *t*-tests as appropriate. Differences were considered significant at *P* < 0.05.

## 3. Results

### 3.1. SLC15A3 Is Induced by Virus Stimulation in Different Cell Types

In order to identify novel genes that are involved in the innate immune response against HSV-1, we analyzed the transcriptomes of HSV-1-stimulated human PBMCs in comparison to PBMCs without HSV-1 stimulation using a previously published data set (GSE60481) [21]. These experiments identified a group of solute carrier (SLC) transporters which were significantly upregulated in HSV-1-stimulated PBMCs as compared to PBMCs without stimulation (Table S3). Among these upregulated SLC genes, SLC15A3 had previously been found to inhibit chikungunya viral replication and to participate in bacterially derived MDP sensing [19, 20]; we therefore selected this gene as our candidate for further investigation.

**Figure 1:**
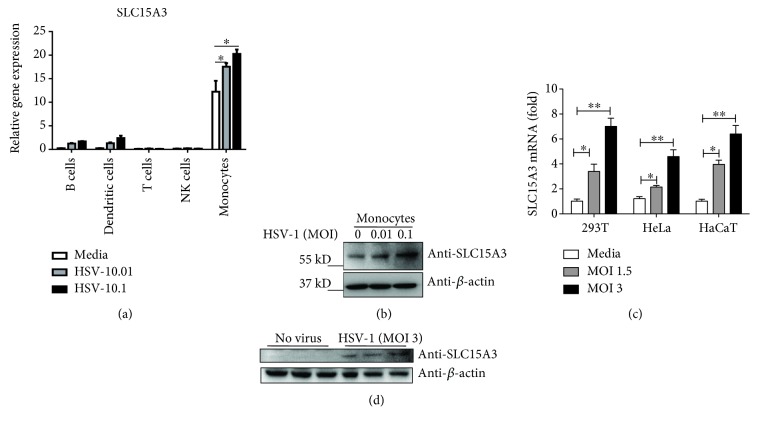
SLC15A3 is induced by viruses' stimulation in different cell types. (a) qRT-PCR analysis of *SLC15A3* mRNA in different types of cells from human PBMCs (*n* = 3) after HSV-1 stimulation for 16 h at indicated MOI. (b) Western blot analysis of SLC15A3 protein in monocytes after HSV-1 stimulation for 16 h at indicated MOI. (c) qRT-PCR analysis of *SLC15A3* mRNA in 293T, HeLa, and HaCaT after HSV-1. (d) Western blot analysis of SLC15A3 protein after HSV-1 stimulation. ^∗^ *P* < 0.05, ^∗∗^ *P* < 0.01 (unpaired *t*-test). Data are representative of three experiments with similar results (mean ± sd in (a) and (c)).

To determine which cell types in human PBMCs are the source of SLC15A3, we isolated T cells, NK cells, B cells, dendritic cells, and monocytes from human PBMCs. We then stimulated these cells with HSV-1 at MOI of 0, 0.01, and 0.1. As shown in Figure 1(a), *SLC15A3* was expressed at the highest levels in monocytes as compared to other cell types, and its expression was upregulated by HSV-1 stimulation. Aside from monocytes, its expression was also upregulated by HSV-1 in B cells and dendritic cells, but the expression levels were much lower than monocytes. SLC15A3 protein in human monocytes was also upregulated by HSV-1 (Figure 1(b)). However, its protein was undetectable in B cells and dendritic cells (data not shown). Additionally, *SLC15A3* mRNA was induced in 293T, HeLa, and HaCaT by HSV-1 (Figure 1(c)). SLC15A3 protein was undetectable at baseline but could be induced by HSV-1 stimulation in 293T cells (Figure 1(d)). Its protein was undetectable in HeLa and HaCaT at both baseline and stimulated conditions (data not shown).

### 3.2. SLC15A3 Is Located in Lysosomes, Late Endosomes, and Peroxisomes

**Figure 2:**
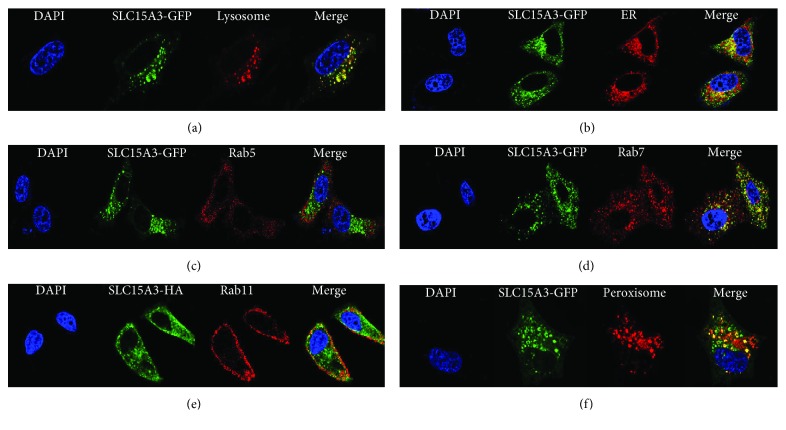
SLC15A3 is located at lysosomes, late endosome, and peroxisome. Confocal microscopy observation of HeLa cells transfected with SLC15A3-GFP (green) and lysosome tracker (a), ER tracker (b), endogenous Rab5 (c), endogenous Rab7 (d), endogenous Rab11 (e), and RFP-PXMP2 (peroxisome tracker) (f).

SLC15A3 was reported to locate in lysosomal membranes [16, 20]. In our current study, we performed comprehensive experiments to determine the subcellular localization of SLC15A3. As shown in Figure 2, SLC15A3-GFP displayed as punctate staining (in green color). Consistent with previous reports, SLC15A3 protein was colocalized with lysosome trackers (Figure 2(a)), but it was not colocalized with ER trackers (Figure 2(b)). Rab5 is an early endosome marker [22], Rab7 is a late endosome marker [23], and Rab11 is a marker for the Golgi apparatus and recycling endosome [24]. We found that SLC15A3 protein was colocalized with Rab7 (Figure 2(d)), but not with Rab5 (Figure 2(c)) and Rab11 (Figure 2(e)), suggesting that it is located in the late endosome. In order to determine whether it is located in peroxisome, we cotransfected SLC15A3-GFP and RFP-PXMP2 into HeLa cells. PXMP2 is a protein that is specifically located in peroxisome [25]. We found that some SLC15A3-GFP proteins were colocalized with RFP-PXMP2, indicating that SLC15A3 is also located in peroxisomes (Figure 2(f)). Taken together, our results demonstrate that SLC15A3 can be located in lysosomes, late endosomes, and peroxisomes.

### 3.3. SLC15A3 Protects Host Cell from DNA Virus HSV-1 Infection

**Figure 3:**
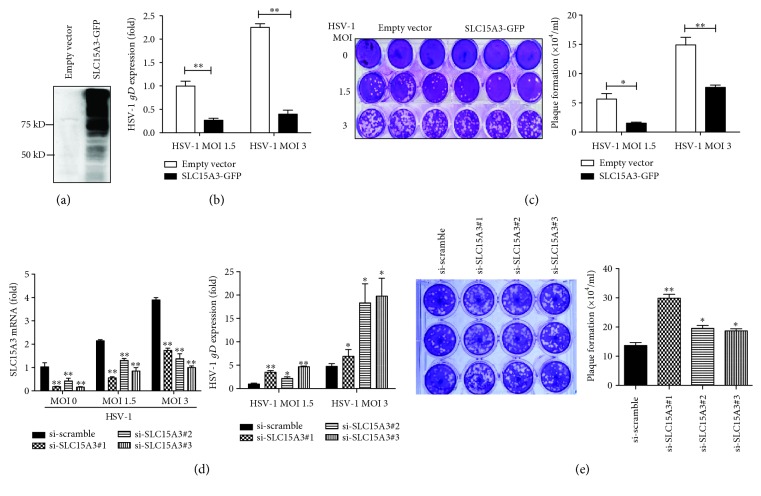
SLC15A3 protects host cells from HSV-1 infection. (a) Western blot analysis of SLC15A3 protein in 293T after overexpression. (b) PCR analysis of HSV-1 *gD* gene abundance in SLC15A3 overexpressed 293T cells. (c) Plaque assays of HSV-1 infectious particles produced in 293T transfected with empty vector and SLC15A3. (d) PCR analysis of *SLC15A3* and HSV-1 *gD* gene expression in 293T transfected with scrambled siRNA and *SLC15A3* siRNA for 24 h then addition of HSV-1 for 24 h. (e) Plaque assays of HSV-1 infectious particles produced in 293T. 293 T cells were transfected with scrambled siRNA and *SLC15A3* siRNA for 24 h then addition of HSV-1 for 24 h. ^∗^ *P* < 0.05, ^∗∗^ *P* < 0.01 (unpaired *t*-test). Data are representative of three experiments with similar results (mean ± sd in (b), (c), (d), (e)).

To address the function of SLC15A3, we overexpressed SLC15A3 and empty vectors in 293T cells, and these cells were then infected with different doses of HSV-1 viruses. HSV-1 replication was evaluated by real-time PCR and viral plaque assays. We found that SLC15A3 protein displayed as a highly polymerized format when it was overexpressed (Figure 3(a)). We used HSV-1 *gD* gene expression to evaluate HSV-1 replication. HSV-1 *gD* gene expression were significantly decreased in SLC15A3-overexpressed 293T as compared to the control compartments (Figure 3(b)). We further used the viral plaque assay to determine the production of infectious HSV-1 particles in SLC15A3-overexpressed 293T as compared to the control cells transfected with empty vectors; SLC15A3-overexpressed 293T cells produced much fewer viral plaques than controls (Figure 3(c)). These results demonstrated that overexpression of SLC15A3 could inhibit HSV-1 replication.

We further used the small RNA interfering method to knock down SLC15A3 in 293T cells and evaluate the effect of deficient SLC15A3 gene expression on HSV-1 replication. As shown in Figure 3(d), SLC15A3 was significantly upregulated by HSV-1 stimulation in 293T cells transfected with empty vectors, and its expression was successfully inhibited by three different siRNA duplexes targeting different RNA regions of SLC15A3. HSV-1 *gD* gene expressions were significantly increased in SLC15A3-deficient cells as compared to cells transfected with scrambled siRNA (Figure 3(d)). Plaque assay results also confirmed that SLC15A3-deficient cells generated significantly increased HSV-1 infectious viral particles than the control cells (Figure 3(e)). These results demonstrated that SLC15A3 deficiency led to enhanced HSV-1 replication. Taken together, we demonstrate that SLC15A3 functions to protect host cell responses against HSV-1 infection.

### 3.4. SLC15A3 Participates in Signaling Pathways That Activate IFN*β* and IL29 by HSV-1

**Figure 4:**
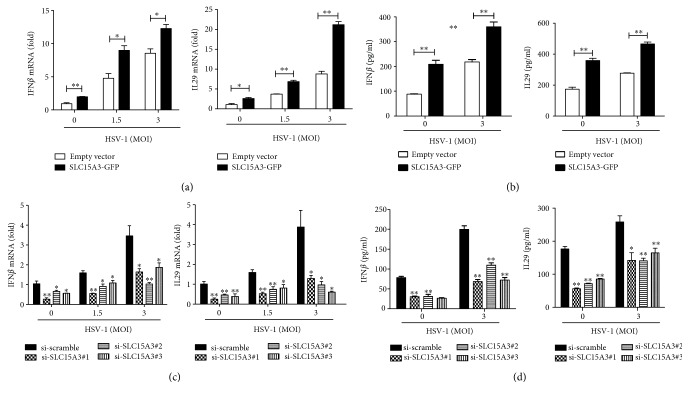
SLC15A3 enhances HSV-1-mediated IL29 and IFN*β* induction. (a) qRT-PCR analysis of IFN*β* and IL29 in 293T transfected with empty vector and SLC15A3-GFP plasmids for 16 h and then incubated with HSV-1 at indicated MOI of HSV-1 for 24 h. (b) ELISA detection of IFN*β* and IL29 in cell culture supernatants collected from 293T transfected with empty vector and SLC15A3-GFP plasmids followed by HSV-1 incubation. (c) qRT-PCR analysis of IFN*β* and IL29 in 293T transfected with scrambled and SLC15A3 siRNA for 24 h and then incubated with HSV-1 at indicated MOI of HSV-1 for 24 h. (d) ELISA detection of IFN*β* and IL29 in cell culture supernatants collected from 293T transfected with scrambled and SLC15A3 siRNA for 24 h and then incubated with HSV-1 at indicated MOI of HSV-1 for 24 h. ^∗^ *P* < 0.05, ^∗∗^ *P* < 0.01 (unpaired *t*-test). Data (mean ± sd) are representative of three experiments with similar results.

Since type I and type III IFNs are major cytokines that protect host cells from viral infection [26], we then investigated whether SLC15A3 was involved in IFN production. As shown in Figure 4(a), upon HSV-1 stimulation, type I IFN, IFN*β,* and type III IFN, IL29, transcripts were significantly increased in SLC15A3-GFP-overexpressed cells compared to cells transfected with empty vector. We also examined IFN*β* and IL29 protein levels in the culture supernatants. Consistently, these proteins were significantly increased in SLC15A3-overexpressed cell culture supernatants in both the presence and absence of HSV-1 stimulation (Figure 4(b)). Conversely, IL29 and IFN*β* gene expressions in SLC15A3-silenced cells were significantly reduced as compared to controls after HSV-1 stimulation (Figures 4(c) and 4(d)). These data suggest that SLC15A3 participates in DNA virus-triggered innate immune responses.

### 3.5. SLC15A3 Interacts with MAVS and STING and Potentiates MAVS- and STING-Mediated IFN Responses

**Figure 5:**
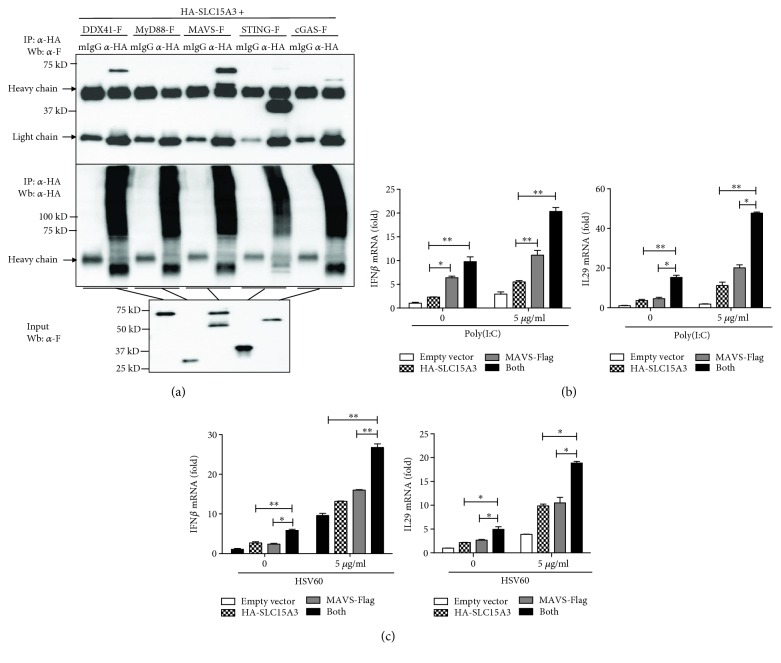
SLC15A3 interacts with MAVS and STING and augments MAVS- and STING-mediated IFN induction. (a) 293T cells were transfected with the indicated plasmids before coimmunoprecipitation and immunoblot analyses were performed with the indicated antibodies. (b) qRT-PCR analysis of IFN*β* in 293T transfected with indicated plasmids for 16 h and then transfected with poly(I:C) for 8 h. (c) qRT-PCR analysis of IFN*β* in 293T transfected with indicated plasmids for 16 h and then transfected with HSV60 for 8 h. ^∗^ *P* < 0.05, ^∗∗^ *P* < 0.01 (unpaired *t*-test). Data (mean ± sd) are representative of three experiments with similar results.

To determine the innate immune signaling pathways involving SLC15A3, we first used immunoprecipitation experiments to investigate SLC15A3-binding partners. The interactions between SLC15A3 and cGAS, MyD88, MAVS, STING, and DDX41 were determined by coimmunoprecipitation. These proteins are the receptors and adaptors for nucleic acid recognition pathways. We found that both MAVS-Flag and STING-Flag proteins were pulled down by HA-SLC15A3 (Figure 5(a)). Additionally, we found that two cytoplasmic DNA sensors, DDX41 and cGAS, could also interact with SLC15A3 (Figure 5(a)). Both DDX41 and cGAS are DNA sensor and use STING as the adaptor [9, 27]. We then investigated whether SLC15A3 regulated MAVS- and STING-mediated IFN responses. Transfected poly(I:C) is an agonist which activates MAVS-mediated signaling pathway, while transfected synthetic double-stranded DNA fragment HSV60 is an agonist activating STING-mediated signaling pathway. We cotransfected SLC15A3 with MAVS and STING, respectively. The cells were then transfected with poly(I:C) and HSV60. We found cotransfection of SLC15A3 and MAVS significantly enhanced IFN*β* and IL29 induction upon intracellular poly(I:C) stimulation (Figure 5(b)), and cotransfection of SLC15A3 and STING also significantly enhanced IFN*β* and IL29 induction upon intracellular HSV60 stimulation (Figure 5(c)), suggesting that SLC38A5 augments MAVS- and STING-mediated IFN responses.

**Figure 6:**
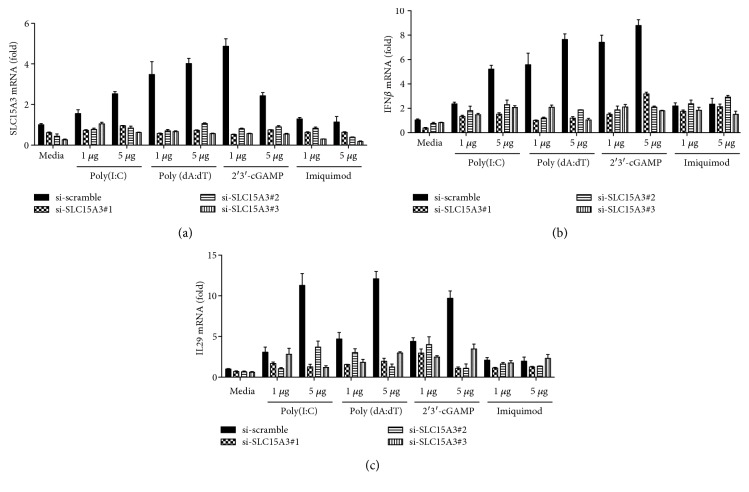
Silencing SLC15A3 in human primary monocytes leads to reduced IFN production by intracellular RNA and DNA agonists. qRT-PCR analysis of (a) SLC15A3, (b) IL29, and (c) IFN*β* in human primary monocytes transfected with scrambled siRNA and SLC15A3 siRNA for 24 h and then incubated with indicated PRR agonists for 8 h. Data (mean ± sd) are representative of three experiments with similar results.

To further validate whether SLC15A3 regulates MAVS- and STING-mediated IFN responses, we silenced SLC15A3 in human primary monocytes then stimulated the cells with poly(I:C), poly(dA:dT), 2′3′-cGAMP, and imiquimod. 2′3′-cGAMP is a STING-specific agonist [28], and imiquimod is a TLR7 agonist [29]. We found that *SLC15A3* was significantly induced by poly(I:C), poly(dA:dT), and 2′3′-cGAMP stimulation, but not by imiquimod, in human monocytes transfected with scrambled siRNA; and its induction was inhibited in cells transfected with *SLC15A3* siRNA (Figure 6(a)). The induction of IFN*β* and IL29 by poly(I:C), poly(dA:dT), and 2′3′-cGAMP was significantly reduced in cells transfected with *SLC15A3* siRNA as compared to cells transfected with scrambled siRNA (Figures 6(b) and 6(c)). TLR7 agonist only slightly induced IFN*β* and IL29, and silencing SLC15A3 did not affect imiquimod-induced responses (Figures 6(b) and 6(c)). These results further suggest that SLC15A3 is involved in both MAVS- and STING-mediated IFN induction.

## 4. Discussion

SLC15A3 has been reported to inhibit chikungunya virus replication when it is ectopically expressed in human STAT1-deficient fibroblasts [19]. However, whether it inhibits the replication of HSV-1 has not been studied. In this study, we demonstrate that SLC15A3 is significantly induced by HSV-1 in different cell types. Overexpression of SLC15A3 inhibits HSV-1 replication, while silencing SLC15A3 leads to enhanced viral replication. Our results therefore extend the spectrum of viruses that SLC15A3 can protect against.

The study by Schoggins et al. simply tested ectopic expression of SLC15A3's effect on chikungunya virus replication [19], but they did not further investigate whether SLC15A3 affected antiviral IFN responses. In our study, we demonstrate that SLC15A3 affects both type I and type III IFN production triggered by HSV-1. Although HSV-1 is an enveloped double-stranded DNA virus, HSV-1 has been found to generate double-stranded RNA [30]. HSV-1 infection causes lytic cell death leading to release double-stranded RNA, which can be endocytosed by nearby cells and transported from late endosome to cytoplasm by late endosome membrane protein SIDT2 [30]. Using double-stranded RNA and DNA agonists, we further found that SLC15A3 is involved in both MAVS- and STING-mediated IFN production, which suggests that SLC15A3 is involved in intracellular double-stranded RNA and DNA sensing. Since SLC15A3 is a late endosome and lysosome-located histidine and dipeptide transporter, we think SLC15A3 may use its transporter function to maintain endosome and lysosome conditions and consequently regulate the activity of DNA and RNA transporters on endolysosomal membrane, thereby affecting cytoplasmic DNA and RNA sensing.

A close family member of SLC15A3, SLC15A4, has been reported to have important functions in TLR7- and TLR9-mediated signaling pathways in plasmacytoid dendritic cells and B cells [31–33]. The molecular mechanisms underlying this regulation are mediated by the transporter function of SLC15A4. SLC15A4 regulates the amino acid composition of lysosome as well as the acidity of lysosome, consequently affecting protease cleavage of TLR7 and TLR9 in lysosomes. This is a crucial step for the activation of these Toll-like receptors [34]. SLC15A4 also regulates mTOR-dependent inflammatory responses in B cells, and deficient SLC15A4 may contribute to the pathogenesis of autoimmune disease [33]. In our study, we do not find the compensatory effect of SLC15A4 to SLC15A3, since silencing SLC15A3 does not increase SLC15A4 gene expression (Figure S1). In our study, we did not use plasmacytoid dendritic cells and B cells which express TLR7 and TLR9; therefore, we cannot exclude the possibility that SLC15A3 has a similar function as SLC15A4. It is of interest for us to investigate the role of SLC15A3 in endolysosomal Toll-like receptors' activation in the future.

Peroxisome is also an organelle that elicits antiviral innate immune responses through membrane-bounded MAVS [35, 36]. Mitochondria located MAVS mediates strong type I IFN*β* responses, while peroxisome-located MAVS mediates type III interferon production [36, 37]. Li et al. reported that not only MAVS is located on the membrane of peroxisome but also STING [25]. In our study, we found SLC15A3 is located on peroxisomes (Figure 2(f)). Moreover, we found that SLC15A3 interacts with MAVS and STING (Figure 5(a)) and enhances MAVS- and STING-mediated type III IFN production (Figures 5(b) and 5(c)). Although the exact mechanism requires further investigation, our data suggest that SLC15A3 may physically bind to MAVS and STING, change their protein conformation, and consequently affect MAVS- and STING-mediated signaling transduction at peroxisome. Additionally, it is possible for SLC15A3 to use its transporter function to regulate peroxisome conditions during infection, then indirectly affecting MAVS- and STING-mediated signal transduction. Aside from the importance of protein trafficking, posttranslational modifications of MAVS and STING, such as ubiquitination, are critical for their signaling complex formation and protein stability [38–40]. For examples, K27-linked polyubiquitination of STING provides a scaffold to recruit TBK1 and IRF3 [41]; iRhom2 can antagonize polyubiquitination of MAVS and STING and consequently maintain the stability of these proteins [42, 43]. It is of interest to investigate whether SLC15A3 is involved in MAVS- and STING-mediated signaling complex formation and posttranslational modifications. Although SLC15A3 pulled down DDX41 and cGAS, the pull down efficiency was much less than STING and MAVS. We therefore think the interaction between SLC15A3 and DDX41 or GAS might be an indirect interaction through STING.

We found that the protein size of SLC15A3 when overexpressed is much bigger than the expected size based on its amino acid composition (Figures 3(a) and 5(a)), suggesting that SLC15A3 protein undergoes detergent-resistant protein modification. However, this phenomenon did not happen to the endogenous SLC15A3 protein in human monocytes and HSV-1-stimulated 293T cells. This discrepancy will need more investigation to delineate.

In summary, our study has revealed that SLC15A3 plays a role in antiviral innate immune responses against HSV-1. In this process, SLC15A3 potentiates MAVS- and STING-mediated IFN production. Our findings open a new avenue of research to study the role of SLC15A3 in innate immune responses against intracellular microbes and related diseases.

## Data Availability

The RNA-sequencing data used to support the findings of this study have been deposited in the Gene Expression Omnibus (GEO) database (Access number GSE60481).

## Abbreviation

ELISA: Enzyme-linked immunosorbent assay
h: Hours
HSV: Herpes simplex virus
IFN: Interferon
MAVS: Mitochondrial antiviral signaling protein
MDA5: Melanoma differentiation-associated gene 5
MDP: Muramyl dipeptide
MOI: Multiplicity of infection
PAMP: Pathogen-associated molecular pattern
PBMC: Peripheral blood mononuclear cell
PHT2: Peptide histidine transporter 2
PRR: Pattern recognition receptor
qRT-PCR: Quantitative reverse transcription polymerase chain reaction
RIG-1: Retinoic acid-inducible gene 1
SLC: Solute carrier
STING: Stimulator of interferon genes
TLR: Toll-like receptor.

## Supplementary Materials

Supplementary Materials Table S1: siRNA sequences of SLC15A3. Table S2: primer sequences. Table S3: solute carrier genes upregulated by HSV-1 in PBMCs. Figure S1: SLC15A4 gene expression in SLC15A3-silenced cells.
