# Supplementary material for: The Solute Carrier Transporter SLC15A3 Participates in Antiviral Innate Immune Responses against Herpes Simplex Virus-1
Source: J Immunol Res. 2018 Jul 5;2018:5214187. doi: 10.1155/2018/5214187 (PMC6057324; doi:10.1155/2018/5214187)
Supplement: Supplementary Materials — Table S1: siRNA sequences of SLC15A3. Table S2: primer sequences. Table S3: solute carrier genes upregulated by HSV-1 in PBMCs. Figure S1: SLC15A4 gene expression in SLC15A3-silenced cells. [file 5214187.f1.docx]

|  | sense（5'-3'） | antisense（5'-3'） |
| --- | --- | --- |
| Si-SLC15A3 #1 | 5’-GCAGCCAAGUGUCCUCUAUTT-3’ | 5’-AUAGAGGACACUUGGCUGCTT-3’ |
| Si-SLC15A3 #2 | 5’-CCGCUUGAUCGACCCUUUATT-3’ | 5’-UAAAGGGUCGAUCAAGCGGTT-3’ |
| Si-SLC15A3 #3 | 5’-GCCAUCAUGGGCAUCUUCUTT-3’ | 5’-AGAAGAUGCCCAUGAUGGCTT-3’ |

Table S1: siRNA sequences of SLC15A3

Table S2 Primer sequences

| *SLC15A3*-F | 5’-TGGCGTTTATTCAGCAGAACA-3’ |
| --- | --- |
| *SLC15A3*-R | 5’-TCTCTGGCCGAGTGTCGTT-3’ |
| *SLC15A4*-F | 5’-CCAGAGCGTTTTCATCACCAA-3’ |
| *SLC15A4*-R | 5’-GCCCACCATGAGACATCTTACA-3’ |
| HSV-1*gD*-F | 5’-CGGCCGTGTGACACTATCG-3’ |
| HSV-1*gD*-R | 5’-CTCGTAAAATGGCCCCTCC-3’ |
| *IFNb*-F | 5’-GCTTGGATTCCTACAAAGAAGCA-3’ |
| *IFNb*-R | 5-ATAGATGGTCAATGCGGCGTC-3’ |
| *IL29*-F | 5’-TCCTAGACCAGCCCCTTCA-3’ |
| *IL29*-R | 5’-GTGGGCTGAGGCTGGATA-3' |
| *β-actin*-F | 5’-CTCCATCCTGGCCTCGCTGT-3’ |
| *β-actin*-R | 5’-GCTGTCACCTTCACCGTTCC-3’ |

Table S3 Solute carrier genes up-regulated by HSV-1 in PBMCs

| Gene Symbol | Mean reads in sham-treated PBMCs(RPKM) | Mean reads in HSV-1 treated PBMCs(RPKM) | Fold(HSV- 1/Sham) |
| --- | --- | --- | --- |
| SLC41A2 | 1.88 | 25.71 | 13.68 |
| SLC38A5 | 11.77 | 105.37 | 8.95 |
| SLC1A3 | 1.95 | 14.14 | 7.25 |
| SLC31A2 | 23.33 | 111.05 | 4.76 |
| SLC6A12 | 2.8 | 12.08 | 4.31 |
| SLC2A6 | 14.9 | 49.7 | 3.34 |
| SLC15A3 | 57.49 | 184.84 | 3.22 |
| SLC25A28 | 47.81 | 133.62 | 2.79 |
| SLC7A11 | 3.53 | 8.57 | 2.43 |
| SLC39A8 | 12.1 | 29.17 | 2.41 |
| SLC1A4 | 6.97 | 16.38 | 2.35 |
| SLC7A7 | 28.72 | 61.87 | 2.15 |
| SLC15A4 | 17.47 | 34.37 | 1.97 |
| SLC43A3 | 18.49 | 36.31 | 1.96 |
| SLC7A5 | 6.2 | 11.61 | 1.87 |
| SLC25A24 | 11.06 | 20.41 | 1.85 |
| SLC43A2 | 33.9 | 62.66 | 1.85 |
| SLC35A4 | 31.3 | 56.97 | 1.82 |
| SLC3A2 | 59.83 | 107.46 | 1.8 |
| SLC35F5 | 9.75 | 16.59 | 1.7 |
| SLC39A1 | 28.7 | 44.35 | 1.55 |
| SLCO3A1 | 14.73 | 21.61 | 1.47 |
| SLC35A5 | 19.62 | 28.43 | 1.45 |
| SLC35G6 | 51.77 | 67.44 | 1.3 |

| A  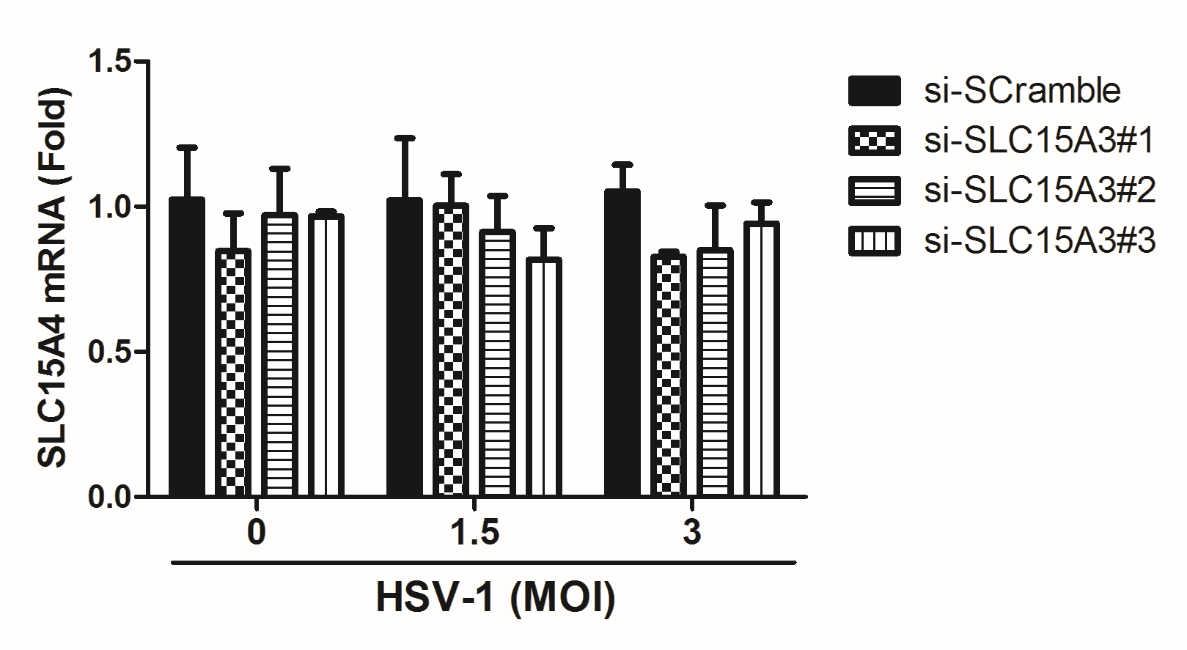  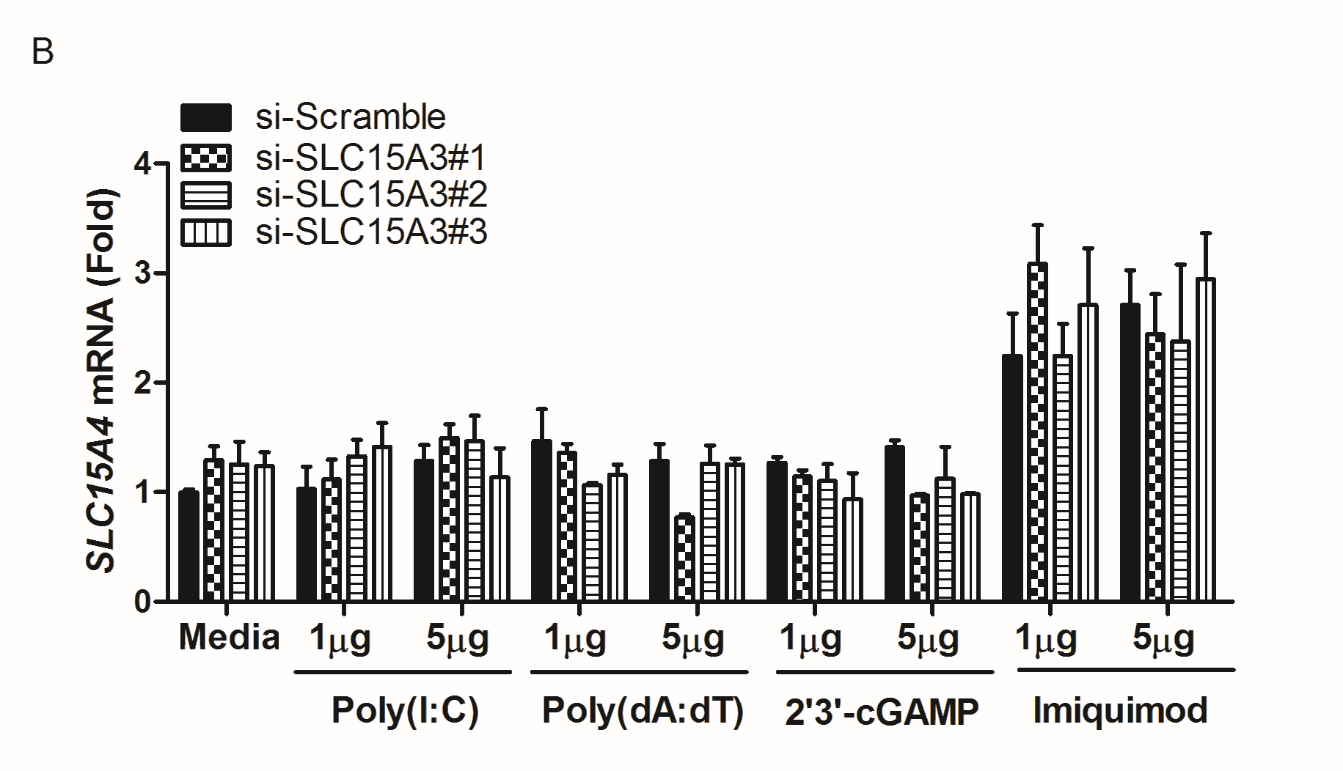 |
| --- |
| Figure S1. *SLC15A4* gene expression in SLC15A3 silenced cells. (A) *SLC15A4* mRNA in 293T cells with indicated siRNA and treatment was evaluated by qRT-PCR; (B) *SLC15A4* in human monocytes with indicated siRNA and treatment was evaluated by qRT-PCR. Data are representative of three experiments with similar results (mean ± s.d.) |
